# Genome-wide analysis of the GH3 family in apple (*Malus × domestica)*

**DOI:** 10.1186/1471-2164-14-297

**Published:** 2013-05-02

**Authors:** Huazhao Yuan, Kai Zhao, Hengjiu Lei, Xinjie Shen, Yun Liu, Xiong Liao, Tianhong Li

**Affiliations:** 1 Department of Fruit Science, College of Agriculture and Biotechnology/Key Laboratory of Stress Physiology and Molecular Biology for Tree Fruits of Beijing, China Agricultural University, 2 Yuanmingyuan West Road, Haidian District, Beijing, 100193, People’s Republic of China

**Keywords:** *Malus sieversii* Roem, Phytohormone, Biotic stress, GH3, DR5, GUS

## Abstract

**Background:**

Auxin plays important roles in hormone crosstalk and the plant’s stress response. The auxin-responsive *Gretchen Hagen3* (*GH3*) gene family maintains hormonal homeostasis by conjugating excess indole-3-acetic acid (IAA), salicylic acid (SA), and jasmonic acids (JAs) to amino acids during hormone- and stress-related signaling pathways. With the sequencing of the apple (*Malus × domestica*) genome completed, it is possible to carry out genomic studies on *GH3* genes to indentify candidates with roles in abiotic/biotic stress responses.

**Results:**

*Malus sieversii* Roem., an apple rootstock with strong drought tolerance and the ancestral species of cultivated apple species, was used as the experimental material. Following genome-wide computational and experimental identification of *MdGH3* genes, we showed that *MdGH3s* were differentially expressed in the leaves and roots of *M. sieversii* and that some of these genes were significantly induced after various phytohormone and abiotic stress treatments. Given the role of *GH3* in the negative feedback regulation of free IAA concentration, we examined whether phytohormones and abiotic stresses could alter the endogenous auxin level. By analyzing the GUS activity of *DR5::GUS*-transformed Arabidopsis seedlings, we showed that ABA, SA, salt, and cold treatments suppressed the auxin response. These findings suggest that other phytohormones and abiotic stress factors might alter endogenous auxin levels.

**Conclusion:**

Previous studies showed that *GH3* genes regulate hormonal homeostasis. Our study indicated that some *GH3* genes were significantly induced in *M. sieversii* after various phytohormone and abiotic stress treatments*,* and that ABA, SA, salt, and cold treatments reduce the endogenous level of axuin. Taken together, this study provides evidence that *GH3* genes play important roles in the crosstalk between auxin, other phytohormones, and the abiotic stress response by maintaining auxin homeostasis.

## Background

Auxin regulates numerous aspects of plant growth and development. To date, auxin has been linked to the control of cell elongation and division, tropic responses to light and gravity, general root and shoot architecture, organ patterning, responses to biotic and abiotic stimuli, vascular development, and growth in tissue culture [1]. Phytohormones are involved in many distinct and/or overlapping processes throughout the life cycle of plants. Auxin facilitates hormonal crosstalk by regulating the expression of auxin-responsive genes [2]. For example, several *ACS* genes, which encode enzymes involved in ethylene biosynthesis, are induced by auxin [3,4]. Auxin homeostasis and the auxin response pathway are regulated by several groups of auxin-responsive genes, including the *Gretchen Hagen3* (*GH3*) family. *Jasmonate resistant 1* (*Jar1*) belongs to the *GH3* gene family in *Oryza sativa* (rice) and is involved in jasmonate signaling [5]. In addition, *AtGH3-5* acts as a bifunctional modulator of both salicylic acid (SA) and auxin signaling during pathogen infection [6]. Auxin also regulates the expression of several genes in the gibberellic acid (GA) biosynthesis pathway [7]. Moreover, auxin homeostasis links growth regulation with stress adaptation responses. For instance, plants subjected to stress conditions exhibit retarded growth, altered patterns of metabolism, and changes in the expression and/or activity of auxin-regulated genes [8,9]. Furthermore, the repression of auxin signaling in Arabidopsis enhances antibacterial resistance [10].

Auxin homeostasis and the auxin response pathway are regulated by several groups of auxin-responsive genes, including the *Gretchen Hagen3* (*GH3*) family [2]. *GH3* was first identified in *Glycine max* (soybean) as an early auxin-responsive gene [11]. To date, *GH3* homologs have been indentified in *Chlorophyta*, *Bryophyta*, *Coniferophyta*, and *Magnoliophyta*[12]. *GH3* family genes are divided into three groups (I, II, and III) based on their sequence similarities and the substrate specificities of their products in Arabidopsis, which harbors 19 GH3 members and one incomplete GH3 protein. [13,14]. Group I GH3 enzymes are JA-amido or SA-amido synthetases [14]. Arabidopsis Group II enzymes were demonstrated to be active on IAA [13,15]. Group III enzymes have only been identified in Arabidopsis to date. Group II *GH3* functions in the negative feedback regulation of IAA concentration. Several Arabidopsis Group II *GH3s* help maintain auxin homeostasis by conjugating excess IAA to amino acids, either for storage or degradation [15]. Members of this gene family are known to be regulated by phytohormones and biotic/abiotic stress factors, including abscisic acid (ABA), SA, JA, drought, cold, salt, pathogen infection, and light [6,16-19]. *GH3*-mediated auxin homeostasis is an essential constituent of the complex network of auxin activity that regulates stress adaptation responses [19]. Recent research has shown that overexpression of *GH3* reduced auxin content and changed plant architecture and plant resistance to biotic and abiotic stress. Overexpression of *TLD1/OsGH3.13* in the rice *tld1-D* mutant resulted in IAA deficiency, dramatic changes in plant architecture, and enhanced drought tolerance [6]. Overexpression of *OsGH3.1* and *OsGH3.8* in rice resulted in reduced auxin content, arrested plant growth and development, abnormal plant morphology, and enhanced pathogen resistance [20,21].

Apple is one of the most widely cultivated fruit trees in the world, and is thus of considerable economic value. Because biotic/abiotic stresses are crucial factors in determining the distribution and yield of apple trees, improving resistance to stresses has been one of the main breeding objectives in apple. *M. sieversii,* an apple rootstock with strong drought tolerance, is an ancestral species of modern apple cultivars that is mainly distributed in the Tianshan Mountains of Central Asia [22,23]. Previous studies in Arabidopsis and rice indicated that *GH3* is involved in the stress response pathway by maintaining auxin homeostasis through conjugating excess IAA to amino acids. In fruit trees, our knowledge of *GH3* genes is mainly limited to their roles in fruit development. *Vitis vinifera* (grapevine) *GH3-1* encodes an IAA-amido synthetase involved in the establishment and maintenance of low IAA concentrations, which enables fruit ripening [24]. Apple *GH3* genes were down-regulated during rapid fruit expansion, consistent with the elevated concentrations of auxin observed at this stage [25].

Synthetic auxin-responsive promoters, such as *DR5*[26], are widely used as experimental readouts for the auxin response and/or auxin levels in planta [27]. *DR5::GUS* contains several copies of a synthetic auxin-responsive element (TGTCTC) fused to a 35S minimum promoter and the GUS encoding sequence [26]. To investigate the role of *GH3* genes in apple, we examined the expression patterns of these genes in *M. sieversii* under biotic and abiotic stress conditions and analyzed whether other phytohormones and abiotic stresses could alter the endogenous distribution of auxin using *DR5::GUS*-transformed Arabidopsis seedlings. We show that *GH3* genes play important roles in the crosstalk between auxin, other phytohormones, and abiotic stress factors in *M. sieversii* by maintaining auxin homeostasis.

## Results

### Genome-wide characterization of the *M. domestica GH3* family

**Figure 1:**
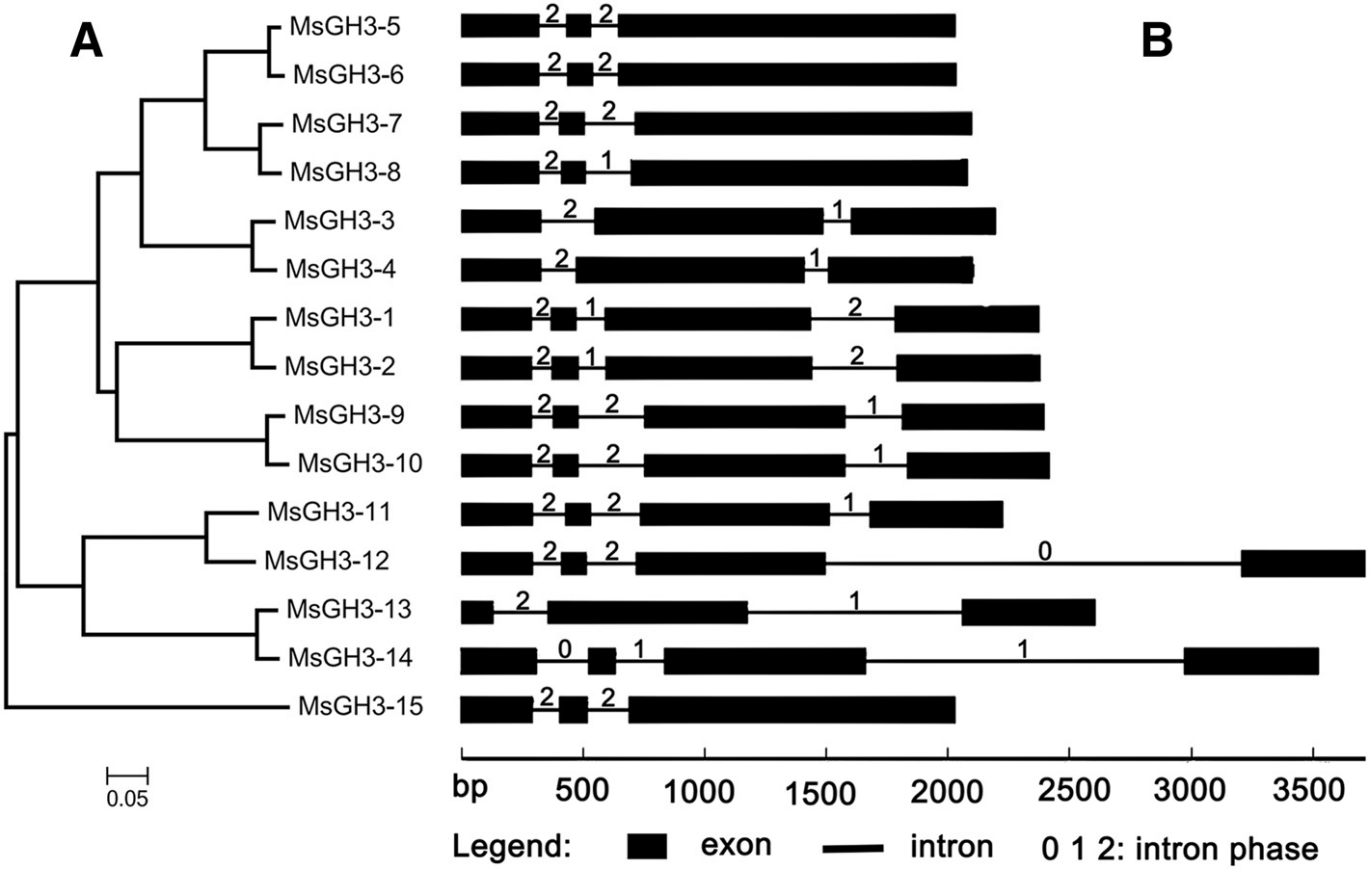
**Phylogenetic analysis and gene structure of apple GH3 members. A** Phylogeny of apple GH3 proteins, generated using MEGA5 (using the neighbor-joining method and a bootstrap test with 1000 iterations). **B** Gene structure of the corresponding apple GH3 proteins, generated by a gene structure display server. The black boxes represent exons and lines represent introns. 0, 1, and 2 represent phase 0, 1, and 2 introns.

**Figure 2:**
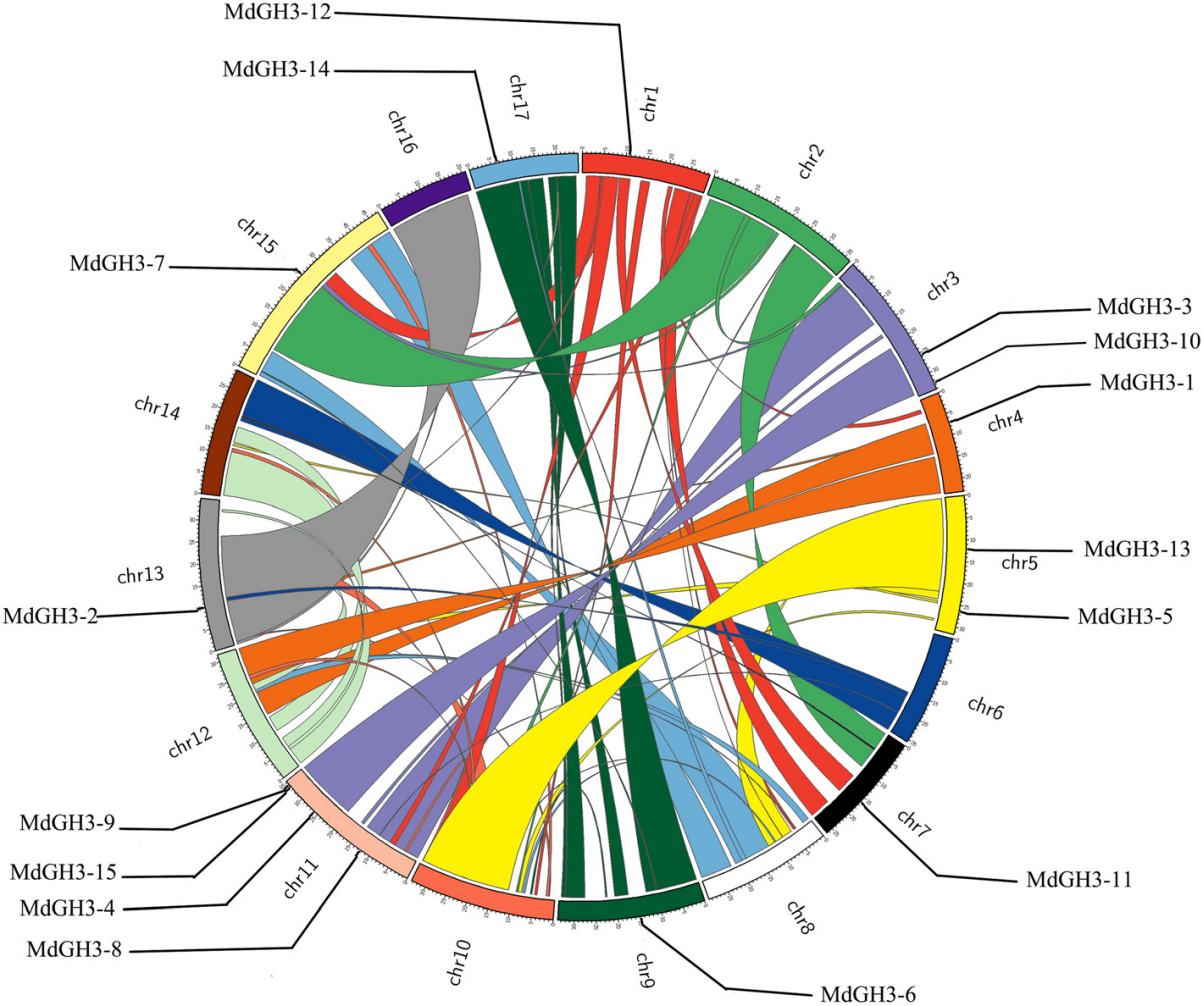
**Mapping of *MdGH3*s and segmental duplication regions on apple chromosomes.** Segmental duplication regions were determined using the SyMAP database. Genes and segmental duplication regions were mapped to the apple chromosomes via the Circos tool. The apple chromosomes were arranged in a circle. Ribbon links represent segmental duplication regions.

From the peptide FASTA file of *M. domestica* genome annotations, we identified 29 candidate GH3 family proteins using the HMMER 3.0 (28 March 2010) program. We disregarded seven of the candidates, as they were below the E-value threshold after the first round of searching. Furthermore, two sequences were repeats of each other, another four sequences were incomplete, with overlapping regions that could be combined into two complete GH3 sequences, and four members were found not to be GH3 family proteins using the BLASTp program at the National Center for Biotechnology Information. Therefore, 15 unique members were present, all of which were confirmed to be GH3 family proteins by the hidden Markov model of the SMART/Pfam tool. Among these full-length coding sequences, four MdGH3 genes (*MdGH3-1, 3, 4,* and *5*) were further confirmed by RT-PCR amplification, cloning, and sequencing (Additional file 1). The MdGH3 polypeptide sequences were all of uniform length (Table 1) and the deduced molecular weight of MdGH3 proteins generally ranged from 64 to 69 kDa. Multiple sequence alignments showed that the MdGH3s were highly conserved (Additional file 2). All of the MdGH3s contained a highly conserved GH3 domain that did not match any other motif in the Pfam database. Pairwise analyses of the full-length protein sequences showed that the overall sequence identities ranged from 26.9% to 96.8% (Additional file 3). Interestingly, MdGH3s formed homeologous pairs, with the sequence identities of homeologous pairs being extremely high; e.g., MdGH3-1/MdGH3-2 (94.4%), MdGH3-3/MdGH3-4 (94.3%), MdGH3-5/MdGH3-6 (96.8%), MdGH3-7/MdGH3-8 (94%), MdGH3-9/MdGH3-10 (95.3%), MdGH3-11/MdGH3-12 (86.9%), and MdGH3-13/MdGH3-14 (94.2%). Then, we examined the phylogenetic relationship and exon–intron organization of apple *GH3* family members. As shown in Figure 1, the homeologous pairs exhibited a close evolutionary relationship and similar gene structures. All of the *MdGH3* genes contained two or three introns, and most had a similar intron phase distribution. We also analyzed the chromosomal location of *MdGH3s*, and found that all 15 *MdGH3s* were distributed on 10 of 17 chromosomes. Four *MdGH3* genes were present on chromosome 11, including a distinct tandem duplicate gene cluster with two tandem genes (*MdGH3-9* and *MdGH3-15*, respectively); two were present on chromosome 3 and also on 5; and one each on chromosomes 1, 4, 7, 9, 13, 15, and 17. None of the *MdGH3* genes were located on chromosomes 2, 6, 8, 10, 12, 14, or 16. Twelve of 15 *MdGH3s,* including homeologous pairs *MdGH3-1/MdGH3-2*, *MdGH3-3/MdGH3-4*, *MdGH3-9/MdGH3-10*, and *MdGH3-11/MdGH3-12,* were mapped on the segmental duplication regions according to information from the SyMAP database (Figure 2).

**Table 1:** **Characteristics of the *GH3 *family in *M. domestica***

**Name**	** GDR^a^**	**Length (aa)^b^**	**ESTs/cDNAs^c^**
MdGH3-1	MDP0000834656	607	GO524295.1
MdGH3-2	MDP0000226842	607	0
MdGH3-3	MDP0000132162	614	0
MdGH3-4	MDP0000402444	614	DT002305.1
MdGH3-5	MDP0000873893	601	CN915524.1 DT043059.1 EB141323.1 DR991447.1 CO898207.1
MdGH3-6	MDP0000209432	601	CN914672.1 EB156443.1 CN908490.1 EB156630.1 EB155796.1 EB144806.1 CV632081.1 CV128791.1 CN995533.2 EB155616.1 EB155541.1 EB156184.1 EB156696.1 EB156293.1 EB156251.1
MdGH3-7	MDP0000872868	599	CN910272.1 CN909305.1 CN907800.1 CN910152.1 CN909148.1 CN910072.1
MdGH3-8	MDP0000612660	599	CN909842.1 CN907795.1 CN907829.1 CN907847.1 CN908348.1 CN908044.1
MdGH3-9	MDP0000204381	596	CV082641.1 CV082778.1
MdGH3-10	MDP0000568498	596	0
MdGH3-11	MDP0000786650	571	CN489575.1
MdGH3-12	MDP0000233483	571	CN912573.1 CN900696.1 GO511018.1 GO528185.1 DY256317.1 CN909380.1 EB141581.1 EB123692.1 GO528114.1 DY255512.1 EB151573.1 EB156721.1 EH034514.1 GO538786.1 EB107177.1 CV631737.1 CV630750.1 EB107168.1 CO899232.1 EB107459.1 GO516821.1 EB107154.1 EB107308.1 EB107425.1 GO535202.1 EB107113.1 CN934444.1 EB107348.1 EB107290.1 DR992460.1 CN933788.1 CN934593.1
MdGH3-13	MDP0000811081	589	GO523947.1 EB109814.1 GO514230.1 CN879199.1 GO510774.1 CN931693.1 GO534539.1 CN445458.1 CN926457.1 DR993803.1
MdGH3-14	MDP0000214081	589	0
	MDP0000238173		
MdGH3-15	MDP0000231245	575	GO522926.1 GO522429.1 GO522817.1

^a^Accession numbers of the proteins at the Genome Database for *Rosaceae*. ^b^Length of the protein in amino acids. ^c^Accession numbers of the ESTs/cDNAs at NCBI.

### *MdGH3* promoter and EST analyses

Cis-acting regulatory DNA elements on both strands of the *MdGH3* promoter were identified using the PLACE web server (http://www.dna.affrc.go.jp/PLACE/), and different DNA elements predicted to be involved in the plant’s response to phytohormones and biotic and abiotic stress were found. The DNA elements included multiple copies of CACGTG (ABA-inducible); TGTCTC (*ARF* (auxin response factor) binding site); ACTTTA (tissue-specific expression and auxin-inducible); CATATG (auxin-inducible); TGACG (IAA/SA-inducible); TTGAC (SA-inducible); AACGTG (JA-inducible); ACGTG and CACATG (drought-inducible); CCGAC (cold/drought-inducible); GAAAAA (salt-inducible); and TGTGA, AAAGAT, and TTGACC (disease-inducible) (see Additional file 4). A BLASTn search of the *M. domestica* EST database (324847 records), which is available at the NCBI webserver (http://blast.ncbi.nlm.nih.gov/), confirmed the transcriptional activity of most *MdGH3s*, but the frequency of ESTs for individual genes varied greatly (Table 1). For example, 32 ESTs were identified for *MdGH3-12* and 15 for *MdGH3-6*, whereas no ESTs had been deposited for *MdGH3-2*, *3*, *10,* and *14* (Table 1).

### Phylogenetic relationship between *M. domestica* and Arabidopsis GH3 family members and *AtGH3* expression analysis using Genevestigator

**Figure 3:**
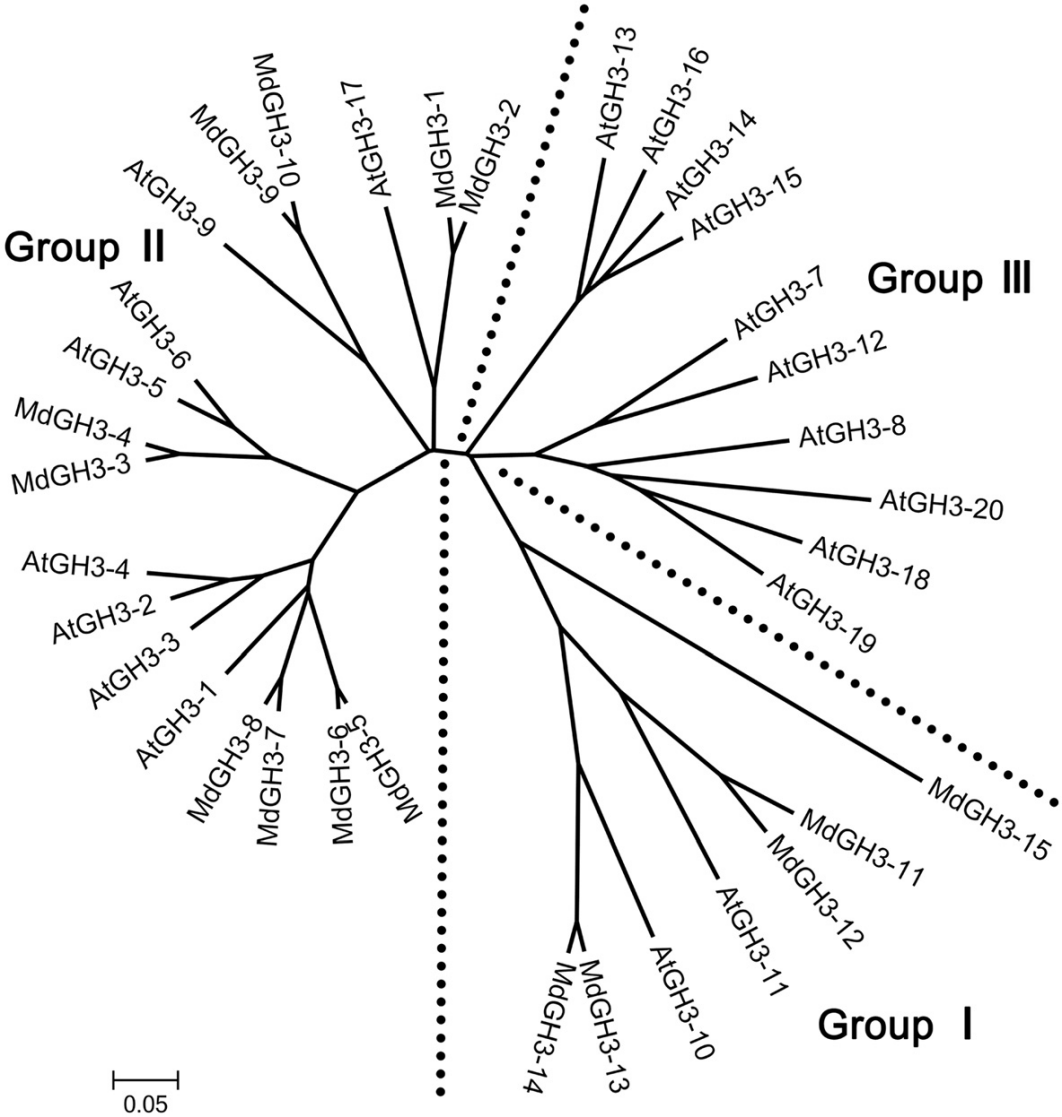
**Phylogenetic tree of *M. domestica *and Arabidopsis GH3 proteins.** The phylogeny was constructed using the neighbor-joining method and a bootstrap test with 1000 iterations, using MEGA5 software, and alignments were generated with ClustalW.

Before analyzing the expression pattern of *MdGH3s,* we performed a preliminary study of *AtGH3* expression. To examine the phylogenetic relationship between *M. domestica* and Arabidopsis GH3 family members, a phylogenetic tree was constructed from alignments of their full-length protein sequences. Whereas AtGH3s can be clustered into three sequence homology groups [13], MdGH3 proteins are only present in two of these (Group I and II; Figure 3). Most of the AtGH3/MdGH3 proteins showed a 1:2 orthologous relationship, such as AtGH3-17 from Arabidopsis and the gene pair from apple, MdGH3-1 and -2. Based on the Arabidopsis microarray data, we found that many of the *AtGH3*s were significantly up-regulated under phytohormone and biotic/abiotic stress treatment (Additional file 5). Most Arabidopsis Group II enzymes were induced by auxin; *AtGH3.1*, *AtGH3.2*, *AtGH3.3*, and *AtGH3.4* were elevated over 10-fold after IAA treatment and *AtGH3.5* and *AtGH3.6* were induced to a lesser extent (2.5-fold to 8-fold). *AtGH3.9* and *AtGH3.17* expression exhibited no remarkable changes in response to auxin. In contrast, no members of the other two groups showed an obvious response to auxin. Only three *AtGH3* members (*AtGH3-3*, *AtGH3-5,* and *AtGH3-6;* all belonging to Group II) were induced over 3-fold within 3 h of ABA treatment. *AtGH3-3* and *AtGH3-4* were slightly induced (under 3-fold) under SA and methyl jasmonate (JA) treatments. Upon cold treatment, *AtGH3-4* expression increased over 7-fold in the green tissue, and *AtGH3-12* was elevated over 10-fold in the root. After drought treatment, only *AtGH3-14* was induced over 6-fold in the root, while the other members had no remarkable response. In response to heat, *AtGH3-3* and *AtGH3-10* increased over 3-fold. Salinity treatment caused a marked induction of *AtGH3-1*, *AtGH3-3*, *AtGH3-4,* and *AtGH3-12* expression in the root. All treatments considered, *AtGH3-3* and *AtGH3-4* responded to most of phytohormone and abiotic stresses, while some of the other *AtGH3* respond to some treatments.

### Differential expression of *MdGH3*s

**Figure 4:**
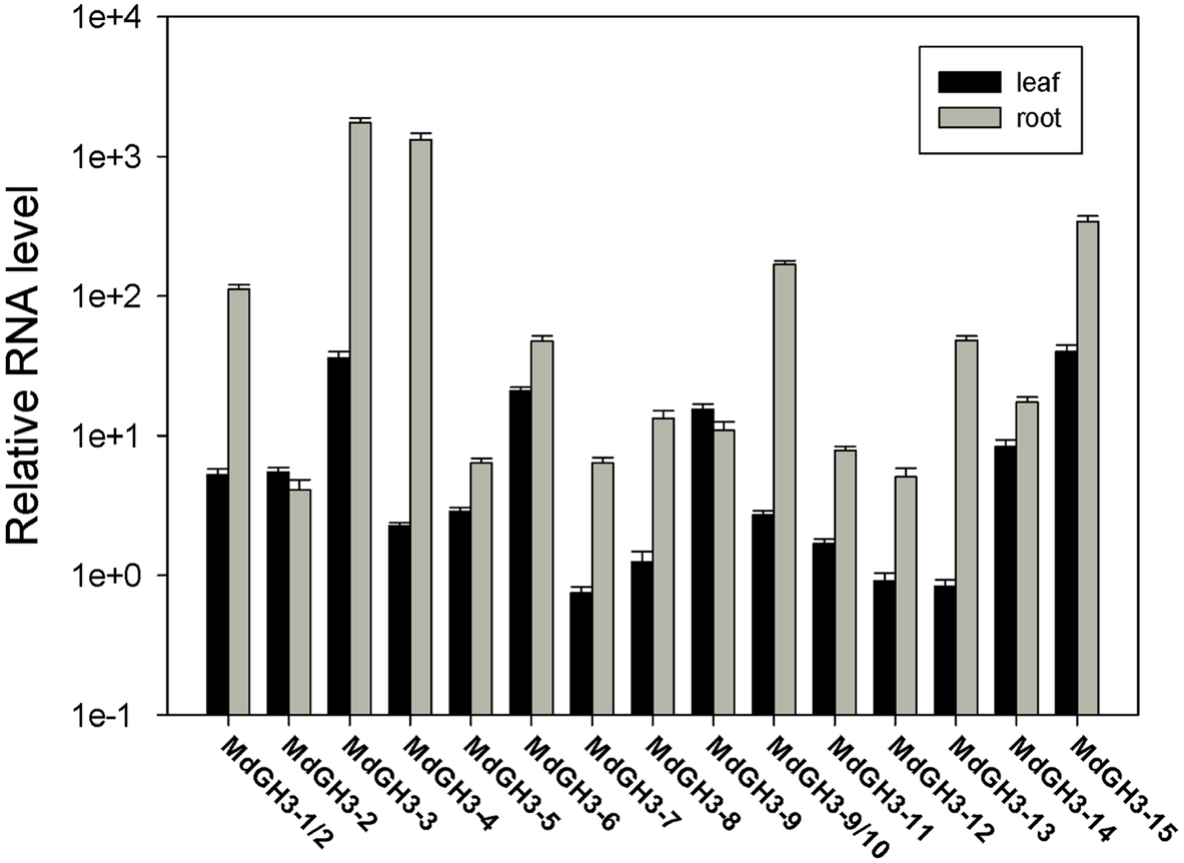
**qRT-PCR analysis of *MdGH3 *genes under normal growth conditions.** Seedlings were grown on Hoagland solution for Data were normalized to the expression level of the apple *HistoneH3* gene. The expression level of apple *HistoneH3* was assumed to be 1e^+5^. Mean expression values were calculated from three independent replicates. Error bars represent the standard error of the mean.

To determine the expression level of *MdGH3*s, qRT-PCR was performed with total RNA isolated from the leaf and root tissues of micropropagated *M. sieversii* plants. Given the high degree of sequence identity between homeologous pairs (Additional file 3), it was challenging to design optimal qRT-PCR primers that were specific for each gene. Since the primers designed for *MdGH3-1* and *MdGH3-10* were predicted to be unable to differentiate between the homeologues, the expression patterns are given with both names (see Additional file 1). The expression level of the apple *HistoneH3* gene was assumed to be 1e^+5^, and was selected as an internal standard in the analysis. qRT-PCR analysis revealed that *MdGH3* genes were differentially expressed in the leaves and roots (Figure 4 and Additional file 6). *MdGH3-7*, *MdGH3-12,* and *MdGH3-13* showed only weak expression in the leaves compared with *MdGH3-3, MdGH3-6, MdGH3-9* and *MdGH3-15. MdGH3-1/2, MdGH3-3* and *MdGH3-4* were strongly expressed in roots under natural growth conditions, compared with MdGH3-2, MdGH3-7, MdGH3-11, and MdGH3-12. Most *MdGH3* genes were more strongly expressed in roots than in leaves, except for *MdGH3-9,* suggesting that *MdGH3* genes are root-specific. In particular, *MdGH3-4* transcripts were over 500-fold higher in roots than in leaves. *MdGH3-3* and *MdGH3-15* showed strong expression in both leaves and roots. However, *MdGH3-2, MdGH3-5, MdGH3-7, MdGH3-11* and *MdGH3-12* expression showed weak expression under normal growth conditions.

### Expression pattern of *MdGH3s* following phytohormone and abiotic stress treatment

**Figure 5:**
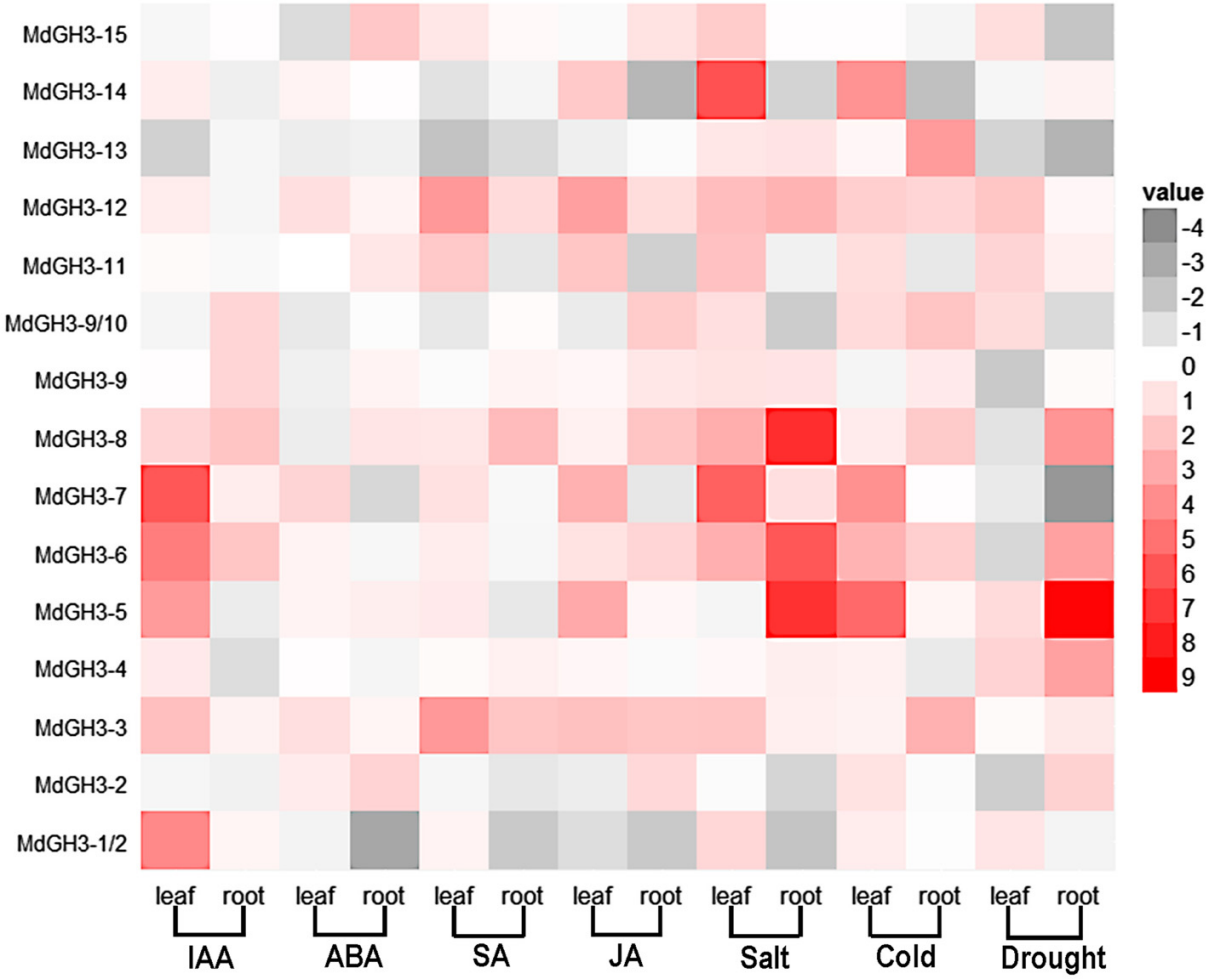
**qRT-PCR analyses of *MdGH3 *genes in plants subjected to various abiotic treatments.** A heat map shows the relative RNA level of *MdGH3* genes in plants under IAA, ABA, SA, JA, drought, cold, and salt treatments, compared to the basal expression level of *MdGH3* genes in plants under normal growth conditions. Data were normalized to apple *HistoneH3* gene expression. Fold difference was designated as the log2 value. The color scale representing the relative RNA level is shown to the right of the heat map.

IAA treatment caused a dramatic induction of *MdGH3-6* and *MdGH3-8* in both the leaves and roots, while the transcript level of *MdGH3-2*, *MdGH3-11*, *MdGH3-13/14*, and *MdGH3-15* was not remarkably changed in either of these tissues upon IAA treatment (Figure 5 and Additional file 7). *MdGH3-1/2, MdGH3-3, MdGH3-5,* and *MdGH3-7* were dramatically upregulated in the leaves only. In contrast, MdGH3-9 and *MdGH3-9/10* were only induced in the roots under IAA treatment. The expression of most *MdGH3* genes was not remarkably changed after ABA treatment in either the leaves or the roots, whereas the expression of *MdGH3-3, MdGH3-7*, and *MdGH3-12* in leaves and *MdGH3-3, MdGH3-8, MdGH3-11,* and *MdGH3-15* in roots was 2 -fold that of the control, suggesting that all of these genes are ABA-responsive. Interestingly, the expression pattern of *MdGH3s* was similar after SA and JA treatment. When SA or JA was applied, *MdGH3-3* and *MdGH3-12* were upregulated in both the leaves and roots, suggesting that MdGH3 proteins participate in the crosstalk between the SA and JA signaling pathways. In contrast, the expression of *MdGH3-1/2, MdGH3-4,* and *MdGH3-13* was not altered upon SA or JA treatment in either leaves or roots. The expression of *MdGH3-7,* and *MdGH3-11* was upregulated only in leaves upon SA or JA treatment. Salinity treatment caused a dramatic induction of *MdGH3-7*(almost 50-fold)*, MdGH3-14* (over 70-fold) in the leaves and *MdGH3-5* (almost 150-fold), *MdGH3-6*(almost 60-fold), *MdGH3-8*(over 160-fold) in the root. Under cold conditions, most *MdGH3* genes were slightly upregulated, whereas the expression of *MdGH3-5* rose 39-fold in the leaves. Most *MdGH3*s showed a slight increase in expression in the leaves or roots. In contrast, *MdGH3-4* and *MdGH3-5* showed increased expression in both the leaves and roots, under drought stress. However, the expression of *MdGH3-5* was strongly induced in leaves (over 460-fold) and roots (over 2-fold) under drought treatment. Interestingly, *MdGH3-5, MdGH3-6, MdGH3-7,* and *MdGH3-8* were markedly induced in the leaves or roots under IAA, salt, cold, and drought treatment, suggesting that these genes might function in the abiotic stress response.

### ABA, SA, salt, and cold treatments suppress the auxin response

Auxin response elements (AuxREs), which consist of a TGTCTC motif and an adjacent or overlapping coupling element, were defined based on the auxin-responsive promoter of the soybean *GH3* gene [28,29]). The finding that native and synthetic promoters containing this element are activated following auxin treatment [29,30] led to the construction of artificial auxin-responsive promoters such as *DR5*[31]. A fusion of the *DR5* promoter with the β-glucuronidase (*GUS)* coding sequence has been frequently used as a maker to monitor endogenous auxin distribution and auxin levels in planta, because the resulting GUS activity coincides with the endogenous IAA distribution [32,33].

**Figure 6:**
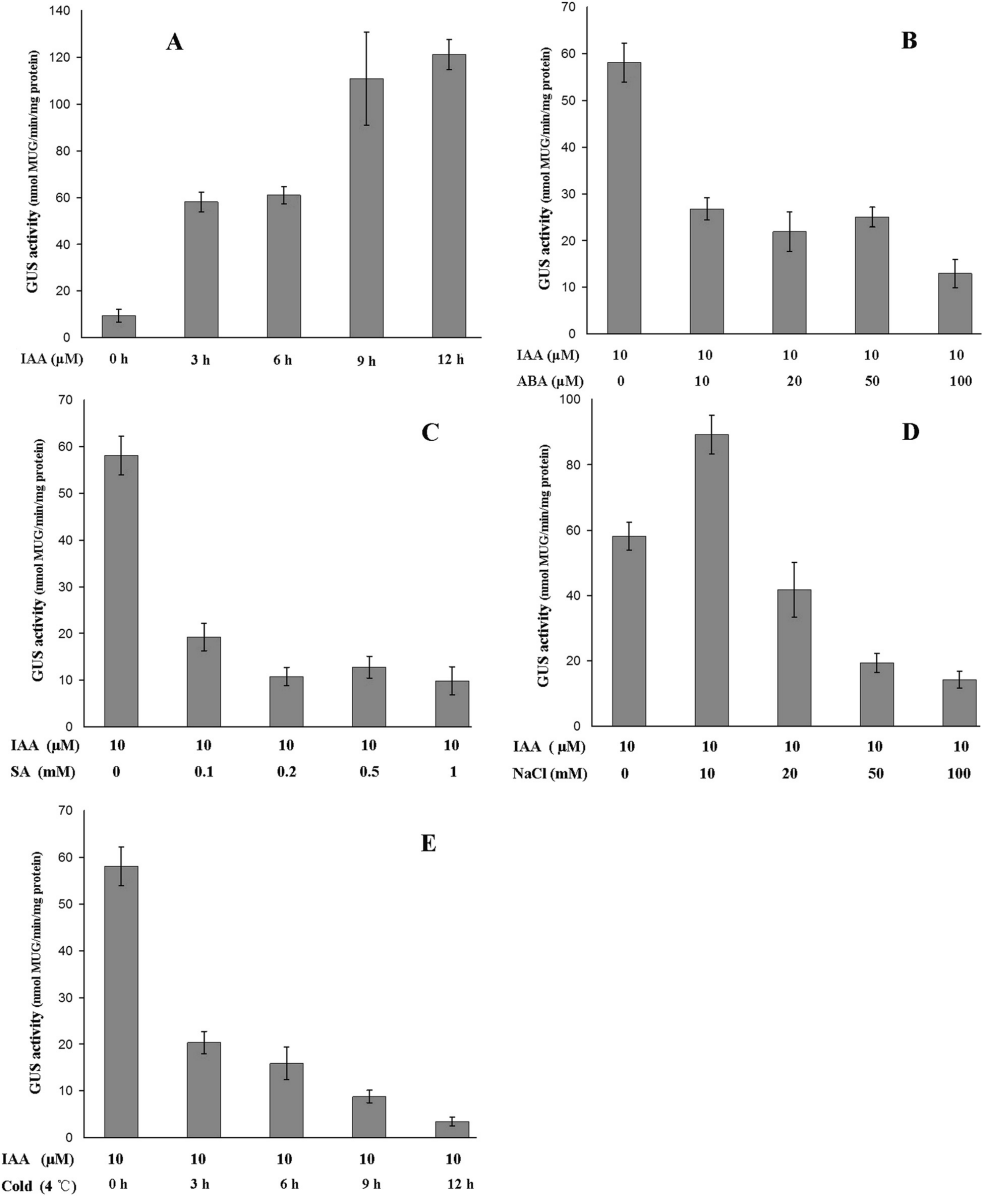
**GUS activity assays of whole *DR5::GUS *transgenic seedlings.** (**A**) *DR5::GUS* seedlings were incubated for an increasing number of hours with 10 μM IAA. (**B**) *DR5::GUS* seedlings were incubated for 3 h with 10 μM IAA and increasing concentrations of ABA. (**C**) *DR5::GUS* seedlings were incubated for 3 h with 10 μM IAA and increasing concentrations of SA. (**D**) *DR5::GUS* seedlings were incubated for 12 h with 10 μM IAA and increasing concentrations of NaCl. (**E**) *DR5::GU*S seedlings were incubated with 10 μM IAA and grown in a growth chamber set to 4°C under a 16/8 h light/dark cycle. The means and SEs of three replicates are shown.

**Figure 7:**
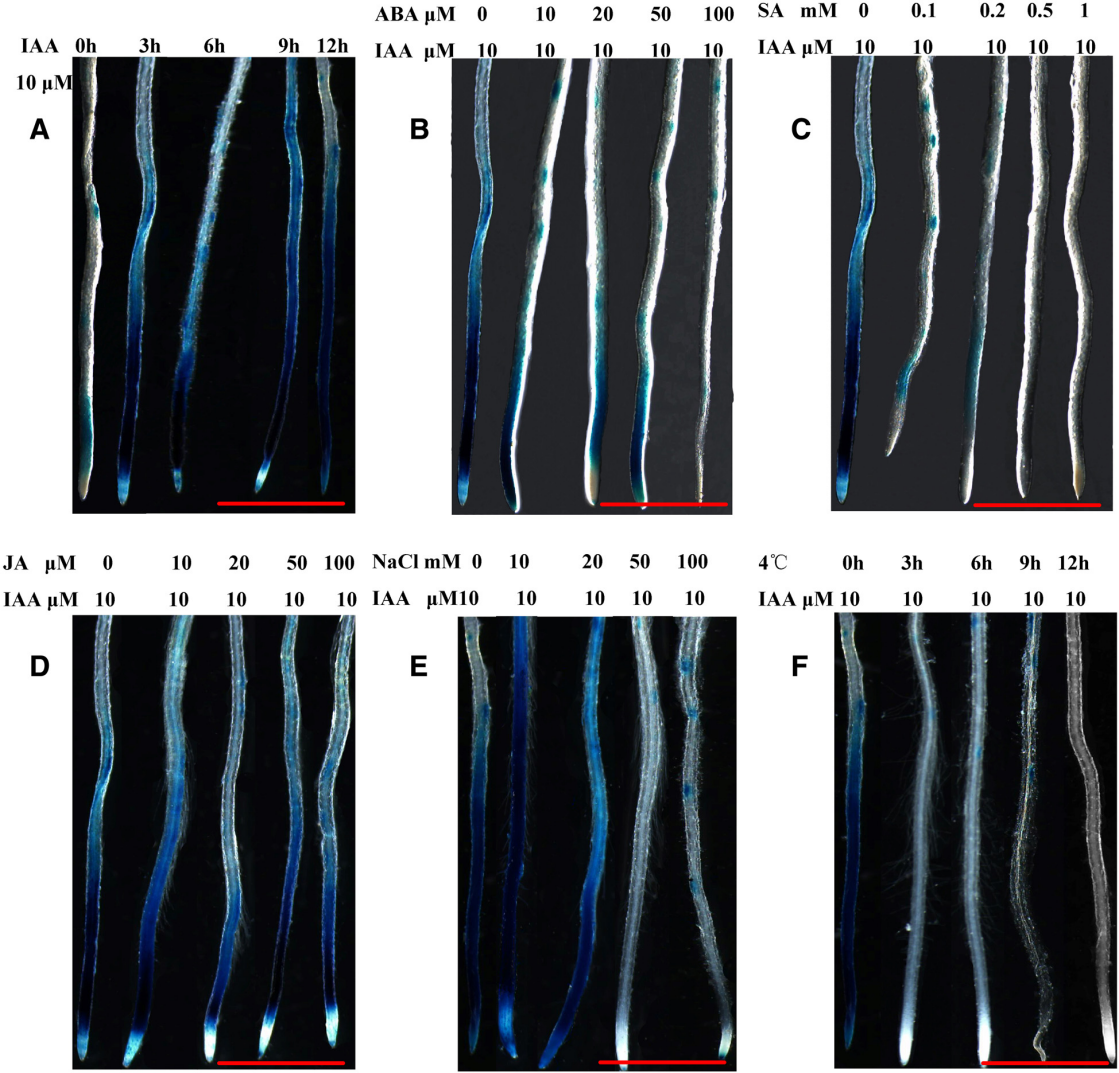
**The effect of IAA, ABA, SA, JA, NaCl, and cold on the expression of *DR5::GUS *in transgenic seedlings.** GUS staining of representative root segments of *DR5::GUS* seedlings after treatment with (**A**) 10 μM IAA for an increasing number of hours, (**B**) 10 μM IAA and an increasing concentration of ABA for 3 h, (**C**) 10 μM IAA and an increasing concentration of SA for 3 h, (**D**) 10 μM IAA and an increasing concentration of JA for 3 h, (**E**) 10 μM IAA and an increasing concentration of NaCl for 12 h, and (**F**) 10 μM IAA and transfer to a growth chamber set at 4°C under the 16/8 h light/dark cycle for an increasing number of hours. Scale bars = 1 cm.

To determine whether other phytohormones and abiotic stresses could alter the endogenous distribution of auxin, we examined the response of the auxin-signaling reporter *DR5::GUS* to various plant hormones and abiotic stresses in Arabidopsis seedlings. As shown in Figure 6A and Additional file 8, treatment with 10 μM IAA induced high levels of GUS activity relative to the control. This result was also observed upon in situ staining for reporter activity in roots, the organs in which the *DR5* promoter is most active (Figure 7A). As shown in Figure 6B-E, ABA, SA, salt, and cold treatments significantly inhibited auxin-mediated expression of this reporter, and the same results were obtained upon in situ staining for GUS activity in roots (Figure 7B, C, E, and F). In the presence of 10 μM ABA or 0.1 mM SA, the increase in GUS activity mediated by treatment with 10 μM IAA was abolished, and this effect was more significant with increasing concentrations of ABA or SA. Likewise, upon incubation with 10 μM IAA at 4°C, GUS activity was significantly lower in the *DR5::GUS* seedlings than in the control. The effect of cold treatment on the suppression of the auxin response was more intense with longer treatments. Exposure to a low concentration of NaCl enhanced the auxin-mediated expression of GUS in *DR5::GUS* seedlings, whereas treatment with a high concentration of NaCl significantly inhibited auxin-mediated GUS expression. However, as shown in Figure 7 D, JA treatment had no effect on the auxin-mediated expression of GUS in *DR5::GUS* seedlings.

## Discussion

Previous studies showed that the expression of *GH3* family genes was regulated by various stress conditions [6,8]. Most studies of *GH3* genes focused on the functional analysis of individual genes in Arabidopsis, rice, and grapevine [18,21,24]. With the availability of the whole genome sequence for apple [34], complete gene families for different classes of genes can be readily identified from genome data based on knowledge of conserved domains. A systematic analysis of the characteristics and phylogeny of apple *GH3* family genes and of their expression patterns upon exposure to phytohormones and abiotic stress would help identify candidates with roles in abiotic/biotic stress responses. *GH3*-mediated auxin homeostasis is an essential constituent of the complex network that underlies crosstalk between auxin signaling and biotic/abiotic stress signaling [6,21]. We investigated whether other phytohormones and abiotic stresses could alter the endogenous auxin distribution using transgenic Arabidopsis seedlings expressing *DR5::GUS*. The results of these analyses will provide the groundwork for further studies on the role of auxin in stress responses.

### The evolution of *GH3* family genes in apple

In this study, we identified 15 putative proteins belonging to the GH3 family in apple (Table 1). The number of GH3 proteins in apple is similar to that in Arabidopsis (10; excluding Group III members, which are unique to Arabidopsis), rice (13), and *Sorghum bicolor* (sorghum) (16) [13,19,35]. Multiple sequence alignments showed that GH3s were highly conserved in apple (Additional file 2), as they are in sorghum [19]. Pairwise analyses with the full-length protein sequences showed that the overall amino acid sequence identities of the full-length GH3s were higher in apple (Additional file 3) than sorghum. *MdGH3* genes emerged in homeologous pairs, with very high sequence similarity. The homeologous pairs had close evolutionary relationships and similar gene structures. Most of the *MdGH3* homeologous pairs were also gene pairs in the segmental duplication regions, which suggests that the apple genome underwent duplication. Indeed, about 60 to 65 million years ago (Mya), the apple genome underwent a whole-genome duplication (GWD) event, which had a great impact on the amplification of members of gene families [34]. Moreover, these results also indicate that the members of an *MdGH3* homeologous pair might have descended from a common ancestor and have similar functions.

*GH3* family genes were divided into three groups (I, II, and III) based on their sequence similarities and the substrate specificities of their products in Arabidopsis, which harbors 19 GH3 members and one incomplete GH3 protein. Group III GH3 enzymes, of which there are nine in Arabidopsis, have only been identified in Arabidopsis to date [13,15]. Our phylogenetic analysis of Arabidopsis and apple GH3 proteins revealed three groups that exhibited distinct orthologous relationships, and no Group III enzymes in apple (Figure 3). Most of the AtGH3s/MdGH3 pairs showed a 1:2 orthologous relationship. Considering the total number of *GH3* family members, genomic duplications were not instrumental in the evolutionary history of the *GH3* family in Arabidopsis [12]. In contrast, genomic duplications had a great impact on the amplification of members of the *GH3* family in apple. This finding also indicates that *MdGH3s* might have begun to diversify as a result of gene duplication. Some AtGH3/MdGH3 pairs exhibited an n:n orthologous relationship, which indicates that the functions of these family members had started to diversify in both Arabidopsis and apple. However, MdGH3-15 has distant orthologous relationships with the other GH3 proteins.

### *GH3* family members involved in plant responses to phytohormones and abiotic stress

To predict the functions of the *MdGH3* genes, we analyzed their promoters and ESTs. The promoters of *MdGH3* contained numerous DNA elements predicted to be induced by phytohormones and biotic and abiotic stresses, suggesting that the expression of *MdGH3*s is regulated by phytohormones and biotic and abiotic stresses (see Additional file 4). Transcriptional activity was confirmed for most of the *MdGH3*s, and the frequency of ESTs implied that genes were expressed at various levels in different tissues (Table 1). Sequence homology analysis represents an important method for predicting gene function. Thus, we examined the expression patterns of *AtGH3*s under phytohormone and biotic/abiotic stress treatment, using the gene expression search engine, Genevestigator. Some of the GH3 family members responded to both phytohormones and abiotic stress in Arabidopsis (Additional file 4)*.*

Our qRT-PCR analysis showed that almost all *MdGH3s* were expressed at a higher level in the roots than in the leaves under normal conditions, indicating that these proteins may be involved in root growth and development (Figure 4). The *ydk1-D* and *dfl1-D* Arabidopsis activation-tagged mutants, which have a T-DNA insertion proximal to a Arabidopsis Group II *GH3* gene, have short primary roots and a reduced number of lateral roots [13,17]. *GH3* was first identified in *Glycine max* as an early auxin-responsive gene [11]. Many of the *GH3* genes in Arabidopsis, soybean, and sorghum can be induced by applying exogenous auxin [13,19,36]. With the exception of *GH3.1*, all Group II *GH3s* in Arabidopsis were demonstrated to be IAA-amido synthetases [13]. In agreement with the *GH3* family expression pattern determined from Arabidopsis microarray data, all GH3 genes of Group II, except *MdGH3-2* and *MdGH3-4*, were dramatically upregulated in apple under IAA treatment, suggesting that the Group II proteins from apple might also be IAA-amido synthetases (Figure 5). However, the expression of most *MdGH3* genes was not markedly changed after ABA treatment. The expression of *MdGH3-3*, *MdGH3-7*, and *MdGH3-12* in the leaves and of *MdGH3-2* and *MdGH3-15* in the roots rose two-fold compared with the control, suggesting that all of these genes are involved in the ABA signaling pathway (Figure 5). SA and JA are known to play key roles in plant defense, and SA- and JA-dependent defense pathways exhibit crosstalk with each other [37-39]. Interestingly, the expression pattern of *MdGH3* genes was similar under SA and JA treatment, which suggests that *MdGH3s* might participate in the crosstalk between SA- and JA-dependent defense pathways (Figure 5). In rice, wild-type seedlings subjected to various abiotic stresses showed a dramatic increase in the transcription of *OsGH3-1*, *OsGH3-8,* and *OsGH3-13* compared with control seedlings [6,40]. In Arabidopsis, *WES1* (*AtGH3/GH3.5*) was strongly induced by ABA and SA treatment and pathogen infections [8]. In sorghum, *SbGH3-1*, *2, 4, 5, 12*, and *13* were markedly induced in leaves upon salt and drought stress treatments [19]. Previous studies showed that *GH3* genes were regulated by various phytohormones and biotic/abiotic stresses. Our analysis of microarray data from Arabidopsis revealed that *AtGH3-3* and *AtGH3-4* were induced by most phytohormone and abiotic stress treatments (Additional file 5). In this study, some *MdGH3s* were markedly induced in response to various phytohormones and biotic/abiotic stress treatments, particularly *MdGH3-5, MdGH3-6, MdGH3-7,* and *MdGH3-8* (Figure 5). Interestingly, our results showed that *MdGH3-5, MdGH3-6, MdGH3-7, MdGH3-8, AtGH3-3,* and *AtGH3-4* were close evolutionary relatives (Figure 3). Taken together, these findings suggest that the *MdGH3* gene family participates in the stress adaptation response, and that *MdGH3-5, MdGH3-6, MdGH3-7,* and *MdGH3-8* may play important roles in this response.

### ABA, SA, salt, and cold treatments suppress the auxin response

A comprehensive study of the effects of SA on auxin signaling based on the Affymetrix ATH1 Gene-Chip for *Arabidopsis thaliana* showed that SA causes the global repression of auxin-related genes, including the *TIR1* receptor gene, resulting in the stabilization of Aux/IAA repressor proteins and the inhibition of auxin responses [41]. An *R2R3*-type *MYB* transcription factor, *MYB96*, regulates the drought stress response by integrating ABA and auxin signals. The MYB96-mediated ABA signals are integrated into an auxin signaling pathway that involves a subset of *GH3* genes encoding auxin-conjugating enzymes [42]. Previous studies indicated that SA and ABA have a negative impact on auxin responses. In addition, the *GH3* gene family can be regulated by phytohormones and biotic/abiotic stress, which act to regulate the auxin pool, effectively modulating auxin responses. We proposed that SA and ABA treatments induce *GH3* expression, which in return reduces the endogenous auxin level. We used *DR5::GUS*, an important tool for localizing regions of auxin responsiveness and/or auxin levels, to test this hypothesis. We detected the effect of various plant hormones and abiotic stress factors on the activity of this reporter in Arabidopsis seedlings. ABA, SA, salt, and cold treatments significantly inhibited the auxin-mediated expression of this reporter, confirming that endogenous auxin levels could indeed be suppressed by these treatments (Figures 6 and 7).

## Conclusion

We performed a genome-wide analysis of the *GH3* gene family in apple, conducted a phylogenetic analysis of the corresponding proteins, and examined their expression profiles in response to phytohormone and abiotic stress treatment. Some *MdGH3* genes were markedly upregulated upon treatment with various phytohormones and biotic/abiotic stresses, especially *MdGH3-5, MdGH3-6, MdGH3-7,* and *MdGH3-8*, which were strongly induced in leaves following IAA, drought, cold, and salt treatment (Figure 5). ABA, SA, salt, and cold treatments caused a sharp decline in IAA concentration (Figures 6 and 7). Given that *GH3* functions in the negative feedback regulation of IAA concentration, we conclude that other phytohormones and abiotic stress factors alter the endogenous distribution of auxin, and that the *GH3* gene family plays an important role in this process by maintaining auxin homeostasis.

## Methods

### Identification of GH3 proteins in the *M. domestica* genome

The genome annotations of *M. domestica* were downloaded from the Genome Database for Rosaceae (http://www.rosaceae.org/node/476). GH3 proteins were identified by Hidden Markov Model (HMM) searches of sequences in the downloaded peptidic FASTA file using the HMMER 3.0 (28 March 2010) program [43] with default parameters. Any sequence that matched the GH3 (PF03321) domain was considered a candidate GH3 protein during the first round. Then, the results were submitted to the Pfam database to confirm that the candidate sequences were apple GH3 proteins. Similarity searches were performed using the BLASTp program at the National Center for Biotechnology Information (http://www.ncbi.nlm.nih.gov/blast/) to confirm the predictions.

### Sequence analysis and chromosomal mapping

The sequence identities were analyzed by pairwise comparisons using the DNASTAR MegAlign 5.01 package. The number and position of exons and introns were determined by comparing the coding sequences (CDSs) with their corresponding genomic DNA sequences, and a map of the gene structure was generated using a gene structure display server [44]. The chromosomal position of each gene was retrieved from the position of the genes stored in the GFF file of the apple genome. Information regarding the segmental duplication regions in the apple genome was retrieved using the SyMAP database [45]. Then, genes and segmental duplication regions were mapped to the apple chromosomes using the Circos tool [46]. Multiple sequence alignments were performed using ClustalW [47]. Phylogenetic analysis was carried out by the neighbor-joining method using MEGA 5 software [48].

### *MdGH3* promoters, EST detection, and *AtGH3* expression analysis

By comparing the CDSs with their corresponding genomic DNA sequences, regions approximately 2,000 bp upstream of the start codon were extracted from the genomic DNA sequences and were designated as promoter sequences. Cis-acting regulatory DNA elements on both strands of the promoter sequences were scanned using the PLACE webserver (http://www.dna.affrc.go.jp/PLACE/). BLASTn was used to perform a search for EST and cDNA sequences against the *M. domestica* EST database (324847 records) using the NCBI webserver (http://blast.ncbi.nlm.nih.gov/). Only hits of the BLASTn search for *MdGH3* showing a bit score of at least 500 were considered to be significant. *AtGH3s* were downloaded from The Arabidopsis Information Resource (TAIR; http://arabidopsis.org). Based on Arabidopsis microarray data from public repositories such as ArrayExpress [49] and GEO [50], we determined the expression patterns of *AtGH3s* under phytohormone and biotic/abiotic stress using the gene expression search engine of Genevestigator [51] (http://www.genevestigator.ethz.ch/).

### Plant materials, growth conditions, and treatments

*Arabidopsis thaliana DR5::GUS*[26] and *M. sieversii* plants were used in this study.

The *DR5::GUS* transgenic plant has been described by Ulmasov [26]. Seedlings were surface sterilized with 10% sodium hypochlorite for 15 min and washed five times with sterile water. Sterilized seeds were cold treated for 4 d at 4°C, germinated on 1/2 Murashige and Skoog medium (MS) with 0.8% (w/v) agar and 3% (w/v) sucrose for 15 days, and transferred to Hoagland solution. Seedlings were grown at 22°C under long-day conditions (16 h light, 8 h darkness). Uniformly developed seedlings of *DR5::GUS* were incubated in water or in solutions containing 10 μM IAA, 10 μM IAA + 10 μM ABA, 10 μM IAA + 20 μM ABA, 10 μM IAA + 50 μM ABA, or 10 μM IAA + 100 μM ABA; 10 μM IAA + 100 μM SA, 10 μM IAA + 200 μM SA, 10 μM IAA + 500 μM SA, or 10 μM IAA + 1000 μM SA; 10 μM IAA + 10 μM MeJA, 10 μM IAA + 20 μM MeJA, 50 μM IAA + 10 μM MeJA, or 10 μM IAA + 100 μM MeJA; or 10 μM IAA + 10 mM NaCl, 10 μM IAA + 20 mM NaCl, 10 μM IAA + 50 mM NaCl, or 10 μM IAA + 100 mM NaCl. For low-temperature treatment, seedlings of *DR5::GUS* incubated with 10 μM IAA were transferred to a growth chamber set at 4°C under long-day conditions.

Micropropagated *M. sieversii* plants were pre-cultured in 1/2 Hoagland nutrient solution for 15 days and then transferred to full-strength Hoagland solution [52]. Plants with heights ranging from 25 to 30 cm were selected for treatments. Uniformly developed seedlings from the liquid culture were treated with 100 μM IAA, 100 μM ABA, 50 μM SA, or 500 μM JA for 3 h, with 150 mM NaCl for 12 h, or at 4°C for 12 h, respectively. Hormones were directly sprayed on the leaf, while NaCl was added to the Hoagland nutrient solution. For drought treatment, seedlings were exposed to air for 12 h. All seedlings were grown at 25°C under a photoperiod of 16 h light/8 h dark, except for those grown at low temperatures.

### RNA extraction and qRT-PCR analysis

Total RNA was extracted using the cetyl trimethyl ammonium bromide (CTAB) method [53]. Genomic DNA was removed from total RNA using RNase-free DNase I (TaKaRa Bio, Shiga, Japan). cDNA was synthesized using an M-MLV Reverse Transcriptase Kit (Promega, Madison, WI, USA) according to the manufacturer’s protocol, and the Oligo(dT) primers and random primers were used in the reverse-transcription reactions. PCR primer pairs were designed using PREMIER Primer 5 software, and evaluated using DNAMAN V6 software (see Additional file 1). Primer sequences were evaluated using the BLAST program to ensure that the primers would allow amplification of unique and appropriate cDNA segments. All real-time PCR assays generated a single band of the expected size, and therefore accurately represented the expression of the queried gene. Melting curve analysis indicated that all the primers generated a single peak. qRT-PCR was performed in the Applied Biosystems 7500 Real-Time PCR System (Applied Biosystems, Foster, CA, USA), using the UltraSYBR Mixture (CWBIO, Beijing, China). PCR amplification conditions for qRT-PCR were 95°C for 10 min, one cycle; and 94°C for 10 s, 60°C for 31 s, 45 cycles. The apple *HistoneH3* gene was selected as an internal standard in the analysis. The relative RNA level of each gene was calculated according to the 2^–ΔΔCT^ method [54]. Each cDNA sample was quantified in triplicate. The data were visualized with the R programming language [55].

### RT-PCR amplification, cloning, and sequencing

Total RNA was extracted using the cetyl trimethyl ammonium bromide (CTAB) method [53] from leaves of *M. sieversii*. cDNA was synthesized using the M-MLV Reverse Transcriptase Kit (Promega, Madison, WI, USA) according to the manufacturer’s protocol. The primer information is given in Additional file 1. RT-PCR amplification conditions were empirically optimized. The PCR products were cloned into the pMD18-T simple vector (TaKaRa Bio, Shiga, Japan) according to the manufacturer’s instructions. The ligated vector DNAs were transformed into *Escherichia coli* DH5α, transformants were plated on LB plates containing 100 μg/mL ampicillin, and isolated plasmid fragments were then sequenced.

### Histochemical analysis of GUS activity

*DR5::GUS* seedlings were incubated with GUS staining solution [56] (1 mM of X-Glu, Gold Biotechnology, St. Louis, Missouri, USA; 100 mM sodium phosphate (pH 7.5), 10 mM EDTA, 0.5 mM potassium ferrocyanide, 0.5 mM potassium ferricyanide, and 0.1% (v/v) Triton X-100) overnight at 37°C. Samples were washed in a graded ethanol series to extract chlorophyll after GUS staining. Images were taken with an OLYMPUS SZX16-DP72 stereo fluorescence microscope.

### Quantitative analysis of GUS activity

After growth in Hoagland solution for 10 days, *DR5::GUS* seedlings were collected and immediately frozen in liquid nitrogen. Total soluble protein was isolated in GUS extraction buffer [56]. The GUS activity of the supernatant was determined using 4-MUG (4-methylumbelliferyl glucuronide) as a substrate. The fluorescence of the GUS-catalyzed hydrolysis reaction product, 4-methylumbelliferone (4-MU), was measured with the TECAN GENios system. Protein concentrations in the supernatant were determined by the Bradford method (1976), using bovine serum albumin (BSA) as a standard. GUS activity was expressed as nmol MUG/min/mg protein. Means ± standard errors (SEs) of three replicates were calculated.

## Competing interests

The authors declare that they have no competing interests.

## Authors’ contributions

HY performed the computational analysis of the *GH3* gene family. Experimental procedures were performed by HY, KZ, HL, XS, YL, and XL TL and HY conceived the project, analysed the data and wrote the paper. All authors read and approved of the final manuscript.

## Supplementary Material

Additional file 1 **Real-time PCR primers used to amplify apple *HistoneH3 * and *MdGH3 *genes, and RT-PCR amplification primers.** Click here for file

Additional file 2 Multiple sequence alignments of full-length MdGH3s. Click here for file

Additional file 3 Pairwise analysis of the overall identities of the full-length MdGH3 protein sequences. Click here for file

Additional file 4 **Promoter analysis of *MdGH3 *genes.** Phytohormone and biotic/abiotic stress response elements are listed. Click here for file

Additional file 5 ***AtGH3 *expression patterns under phytohormone and biotic/abiotic stress.** Click here for file

Additional file 6 **Real-time PCR analysis of *MdGH3 *expression in the leaves and roots of *M. sieversii.*** Click here for file

Additional file 7 **Real-time PCR analysis of *MdGH3 *expression in the leaves and roots of plants subjected to phytohormone and abiotic stress.** Click here for file

Additional file 8 **GUS activity assays of whole *DR5::GUS *transgenic seedlings subjected to phytohormone and abiotic stress treatments.** Click here for file
