# Supplementary material for: Genome-wide analysis of the GH3 family in apple (Malus × domestica)
Source: BMC Genomics. 2013 May 2;14:297. doi: 10.1186/1471-2164-14-297 (PMC3653799; doi:10.1186/1471-2164-14-297)
Supplement: Additional file 1 — Real-time PCR primers used to amplify apple HistoneH3 and MdGH3 genes, and RT-PCR amplification primers. [file 1471-2164-14-297-S1.doc]

Real-time PCR primers used to amplify apple *HistoneH3* and *MdGH3* genes, and RT-PCR amplification primers

| gene | Forward primer (5′-3′) | Reverse primer (5′-3′) |
| --- | --- | --- |
| Real-time PCR primers for apple *HistoneH3* gene, *MdGH3* genes | | |
| *HistoneH3* | TGAAGAAGCCCCACAGATA | GTCAGTCTTGAAGTCCTGCG |
| *MdGH3-1/2* | TATAATCTCCTTGTGCCTGTGATG | GCGGAAGTTACTGCTCTTGTAG |
| *MdGH3-2* | GTAGAGCGAGCTTTGAACCGA | CTGCTGTACTTCATACGCATTTGT |
| *MdGH3-3* | CTCTCATACAGTTTCAAGCCAATCT | GGGTGAGTATTTCAGCGAGGAC |
| *MdGH3-4* | AACATTGATTAGCAAGGGATTGAA | AAATGGGCAGTAGTTACTCTTTCC |
| *MdGH3-5* | GTCAGCCCATCTTTACCGCA | CATCATCAACAAACCCACATCT |
| *MdGH3-6* | CGATTAACCAGTACAAGGTGCC | CAGTCCTAACCCATAAATTATCCAA |
| *MdGH3-7* | CCATCATGGAACTTCTTGACTCTAG | TTACATATCACCCATTGAAATACCC |
| *MdGH3-8* | CACCATAACCTAGATCATATCTCCCT | CTGGTCGTCTCCTCTATGAACTGA |
| *MdGH3-9* | CAATCAATATAAAACGCCGAGG | TTAGTTCAGTAGCAGGAAAATAAAGG |
| *MdGH3-9/10* | ACGCGGATACGTCATGTCTAC | CAAGTTCCTCTTCGACAGCAAT |
| *MdGH3-11* | CTCTCCCTCAGACTTCGATTCAC | AACTCGTCCAACTTCTCCAACAT |
| *MdGH3-12* | TCCATAGTTTGATTTGTTGGTATTG | GAACCCTCTCAGCATCCTTTG |
| *MdGH3-13* | ATAAGTGAAGGATAAAAACGACAGC | ATGGTAGTGGTTGTGCTTGTTGTAT |
| *MdGH3-14* | ACTAGAATGTGAGAAGCCAACTTTG | AAGTGTCCCTGCTATTTTGTTTCT |
| *MdGH3-15* | TGAGGGGAAGAGTTGGAGTG | AGCATTCCGAAGCGAAGTAG |
| RT-PCR amplification primers | | |
| *MdGH3-1* | CAGTGAGTGACGTCCTAGAATCG | CCAAATCCAACATACACAATGAGAG |
| *MdGH3-3* | ACACCATGCCTGAAGCACCGAAG | TGGACCTCCATGAAAGCGGCTT |
| *MdGH3-4* | ACCATCATCTCTTTTTCGTCACAC | CCATCCCCCTTCCATTTTTATT |
| *MdGH3-5* | CTAATCTTCAAATGGCTGTCG | TTCTTTCCGCAACTCAAT |
