# Supplementary material for: Genome-wide analysis of the GH3 family in apple (Malus × domestica)
Source: BMC Genomics. 2013 May 2;14:297. doi: 10.1186/1471-2164-14-297 (PMC3653799; doi:10.1186/1471-2164-14-297)
Supplement: Additional file 2 — Multiple sequence alignments of full-length MdGH3s. [file 1471-2164-14-297-S2.docx]

**
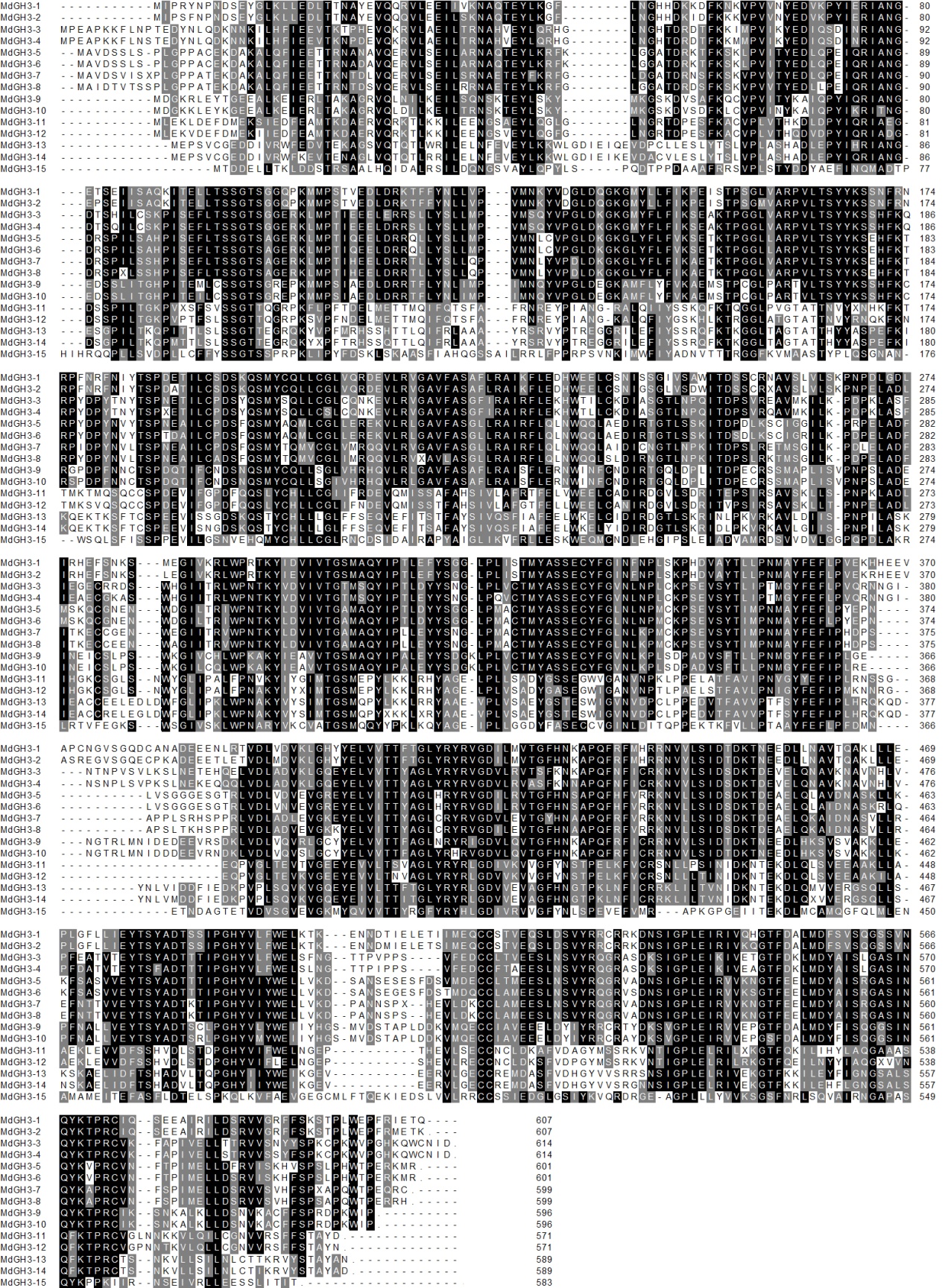
**Multiple sequence alignments of full-length MdGH3s. Identical and conserved residues (present in more than 50% of aligned sequences) are highlighted in black and gray, respectively. Alignments were generated using ClustalW.
