# Supplementary material for: Genome-wide analysis of the GH3 family in apple (Malus × domestica)
Source: BMC Genomics. 2013 May 2;14:297. doi: 10.1186/1471-2164-14-297 (PMC3653799; doi:10.1186/1471-2164-14-297)
Supplement: Additional file 3 — Pairwise analysis of the overall identities of the full-length MdGH3 protein sequences. [file 1471-2164-14-297-S3.doc]

Pairwise analysis of the overall identities of the full-length MdGH3 protein sequences.

|  | Md  GH3-1 | Md  GH3-2 | Md  GH3-3 | Md  GH3-4 | Md  GH3-5 | Md  GH3-6 | Md  GH3-7 | Md  GH3-8 | Md  GH3-9 | Md  GH3-10 | Md  GH3-11 | Md  GH3-12 | Md  GH3-13 | Md  GH3-14 | Md  GH3-  15 |
| --- | --- | --- | --- | --- | --- | --- | --- | --- | --- | --- | --- | --- | --- | --- | --- |
| Md  GH3-1 | *** | 94.4 | 56.3 | 56 | 54.3 | 54.2 | 53.2 | 53.5 | 58.5 | 58.1 | 36.9 | 37.4 | 33.9 | 33.4 | 30.3 |
| Md  GH3-2 |  | *** | 55.9 | 55.4 | 54.2 | 54 | 53.3 | 53.5 | 58.5 | 57.6 | 37.1 | 37.8 | 34.1 | 33.2 | 30.3 |
| Md  GH3-3 |  |  | *** | 94.3 | 65.3 | 64.3 | 64.8 | 64.7 | 54.3 | 54.4 | 36.4 | 37.4 | 35.4 | 34.7 | 29.1 |
| Md  GH3-4 |  |  |  | *** | 64.6 | 64.1 | 64.7 | 64.7 | 53.9 | 54.1 | 36.4 | 37.2 | 34.7 | 34.4 | 29 |
| Md  GH3-5 |  |  |  |  | *** | **96.8** | 80 | 81 | 52.9 | 52.8 | 36.5 | 36.4 | 33.2 | 33.4 | 29.3 |
| Md  GH3-6 |  |  |  |  |  | *** | 79.3 | 80.5 | 52.6 | 52.3 | 36.4 | 36.7 | 32.4 | 32.4 | 29 |
| Md  GH3-7 |  |  |  |  |  |  | *** | 94 | 52.8 | 52.1 | 36.2 | 37.2 | 32.7 | 33.4 | 28.4 |
| Md  GH3-8 |  |  |  |  |  |  |  | *** | 52.8 | 52.3 | 37.2 | 37.2 | 33.2 | 33.2 | 29.3 |
| Md  GH3-9 |  |  |  |  |  |  |  |  | *** | 95.3 | 37.1 | 37.6 | 34.9 | 33.7 | 27.6 |
| Md  GH3-10 |  |  |  |  |  |  |  |  |  | *** | 36.7 | 36.7 | 34.6 | 33.2 | **26.9** |
| Md  GH3-11 |  |  |  |  |  |  |  |  |  |  | *** | 86.9 | 52.3 | 52.6 | 31.8 |
| Md  GH3-12 |  |  |  |  |  |  |  |  |  |  |  | *** | 52.1 | 51.7 | 31.6 |
| Md  GH3-13 |  |  |  |  |  |  |  |  |  |  |  |  | *** | 94.2 | 29.5 |
| Md  GH3-14 |  |  |  |  |  |  |  |  |  |  |  |  |  | *** | 30 |
| Md  GH3-15 |  |  |  |  |  |  |  |  |  |  |  |  |  |  | *** |

The highest and lowest identities are marked in bold.
