# Supplementary material for: Genome-wide analysis of the GH3 family in apple (Malus × domestica)
Source: BMC Genomics. 2013 May 2;14:297. doi: 10.1186/1471-2164-14-297 (PMC3653799; doi:10.1186/1471-2164-14-297)
Supplement: Additional file 5 — AtGH3 expression patterns under phytohormone and biotic/abiotic stress. [file 1471-2164-14-297-S5.docx]

*
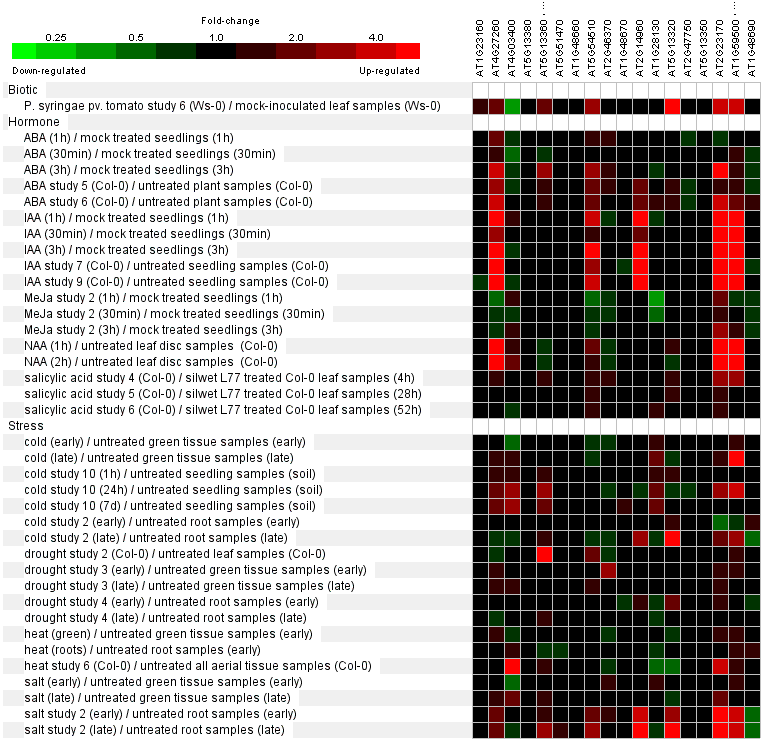
AtGH3* expression patterns under phytohormone and biotic/abiotic stress. The gene expression search engine Genevestigator (http://www.genevestigator.ethz.ch/), coupled with *Arabidopsis* microarray data, was used to generate these results. The color scale represents the relative RNA level, as indicated. The AGI gene names of *Arabidopsis* GH3 proteins are: AtGH3-1, AT2G14960; AtGH3-2/YDK1, AT4G37390; AtGH3-3, AT2G23170; AtGH3-4, AT1G59500; AtGH3-5, AT4G27260; AtGH3-6/DFL1, AT5G54510; AtGH3-7, AT1G23160; AtGH3-8, AT5G51470; AtGH3-9, AT2G47750; AtGH3-10, AT4G03400; AtGH3-11/FIN219/JAR1, AT2G46370; AtGH3-12, AT5G13320; AtGH3-13, AT5G13350; AtGH3-14, AT5G13360; AtGH3-15, AT5G13370; AtGH3-16, AT5G13380; AtGH3-17, AT1G28130; AtGH3-18, AT1G48660; AtGH3-19, AT1G48670; and AtGH3-20, AT1G48690. AtGH3-2/YDK1, AT4G37390, AtGH3-14, AT5G13360, and AtGH3-15, AT5G13370 were detected using the same microarray probes.
