# Supplementary material for: Genome-wide analysis of the GH3 family in apple (Malus × domestica)
Source: BMC Genomics. 2013 May 2;14:297. doi: 10.1186/1471-2164-14-297 (PMC3653799; doi:10.1186/1471-2164-14-297)
Supplement: Additional file 6 — Real-time PCR analysis of MdGH3 expression in the leaves and roots of M. sieversii. [file 1471-2164-14-297-S6.doc]

*Real-time PCR analysis of MdGH3 expression in the leaves and roots of* M. sieversii

| Gene | leaf | Error bars | root | Error bars |
| --- | --- | --- | --- | --- |
| MdGH3-1/2 | 5.259 | 0.231 | 111.473 | 9.234 |
| MdGH3-2 | 5.523 | 0.421 | 4.084 | 0.768 |
| MdGH3-3 | 36.013 | 1.872 | 1731.632 | 42.592 |
| MdGH3-4 | 2.263 | 0.124 | 1310.323 | 49.600 |
| MdGH3-5 | 2.874 | 0.193 | 6.363 | 0.550 |
| ­MdGH3-6 | 20.876 | 1.210 | 47.4 | 2.405 |
| MdGH3-7 | 0.751 | 0.017 | 6.384 | 0.406 |
| MdGH3-8 | 1.252 | 0.235 | 13.276 | 1.775 |
| MdGH3-9 | 15.424 | 1.376 | 10.823 | 1.663 |
| MdGH3-9/10 | 2.714 | 0.175 | 169.237 | 9.563 |
| MdGH3-11 | 1.682 | 0.148 | 7.867 | 0.544 |
| MdGH3-12 | 0.912 | 0.023 | 5.077 | 0.788 |
| MdGH3-13 | 0.837 | 0.045 | 48.257 | 3.479 |
| MdGH3-14 | 8.392 | 0.964 | 17.275 | 1.570 |
| MdGH3-15 | 39.875 | 2.432 | 342.526 | 22.257 |
