# Supplementary material for: Genome-wide analysis of the GH3 family in apple (Malus × domestica)
Source: BMC Genomics. 2013 May 2;14:297. doi: 10.1186/1471-2164-14-297 (PMC3653799; doi:10.1186/1471-2164-14-297)
Supplement: Additional file 8 — GUS activity assays of whole DR5::GUS transgenic seedlings subjected to phytohormone and abiotic stress treatments. [file 1471-2164-14-297-S8.doc]

GUS activity assays of whole *DR5::GUS* transgenic seedlings subjected to phytohormone and abiotic stress treatments( GUS activity was expressed as nmol MUG/min/mg protein).

| IAA (10 μM) | 3 h | 6 h | 9 h | 12 h |
| --- | --- | --- | --- | --- |
| GUS activity | 58.091 | 60.992 | 110.889 | 121.162 |
| Error bars | 4.201 | 3.800 | 20.003 | 6.489 |
| IAA (10 μM) | + ABA (10 μM) | + ABA (20 μM) | + ABA (50 μM) | + ABA (100 μM) |
| GUS activity | 26.778 | 21.918 | 25.039 | 12.971 |
| Error bars | 2.390 | 4.203 | 2.108 | 3.019 |
| IAA (10 μM) | + SA (0.1 mM) | + SA (0.2 mM) | + SA (0.5 mM) | + SA (1 mM) |
| GUS activity | 19.226 | 10.783 | 12.764 | 9.898 |
| Error bars | 2.930 | 1.923 | 2.300 | 2.980 |
| IAA (10 μM) | + NaCl (10 mM) | + NaCl (20 mM) | + NaCl (50 mM) | + NaCl (100 mM) |
| GUS activity | 89.217 | 41.745 | 19.316 | 17.234 |
| Error bars | 8.278 | 12.234 | 2.829 | 2.101 |
| IAA (10 μM) | + 4℃ ( 3 h) | + 4℃ (6 h) | + 4℃ (9 h) | + 4℃ (12 h) |
| GUS activity | 20.348 | 15.903 | 8.789 | 3.455 |
| Error bars | 2.394 | 3.491 | 1.374 | 0.923 |
| IAA (10 μM) | CK (IAA 0 h) |  | | |
| GUS activity | 9.378 |  | | |
| Error bars | 2.800 |  | | |
